# Proceedings of Virginia Dental Association

**Published:** 1875-03

**Authors:** 


### ARTICLE II.

### *Virginia Dental Association.*

#### First Day.

This Association convened December 15th, 1874, in its fifth annual session at Association Hall, Dr. W. W. H. Thackston, of Farmville, president; Dr. George F. Keesee, secretary.

On calling the roll, it was ascertained that nearly all of the city dentists were present, with a fair representation from other parts of the State.

The Committee on Membership reported favorably on the following names, and they were unanimously elected: Drs. W. L. Cowardin, W. L. Burton, C. A. Mercer, Richmond; George H. Cooke, Louisa, and Thomas L. Sydnor, Salem.

Dr. R. N. Hudson, having retired from the practice of dentistry, was elected an honorary member.

An invitation was extended by Drs. John G. and George G. Wayt, to the members to witness an electric engine, and the application of steam to the Morrison engine, at their office, which was gratefully accepted.

The report on dental histology and microscopy, was read by Dr. Wood, accompanied by diagrams, and was an interesting and instructive document, and from which the writer deduced the following: That the dental pulp is composed of fibrillæ more or less branching. The organ extends to, surrounds, and closely invests the pulp. Hard dentine is calcified dentine. The portion remaining uncalcified we call dental fibrillæ. There is no inter-fibrillæ substance whatever. Fresh dentine, therefore, has no tubuli, but has uncalcified fibrillæ. That the difference between dentine and the fibrillæ is simply one of mineral element. Enamel and dentine are not vitalized tissues as far as the calcified portions are concerned. Enamel proper and dentine proper are amenable to physical and chemical laws only, and therefore any sensitiveness in dentine is in virtue of its fibrillæ and of their organic continuity with the terminal nerve-fibres in the pulp.

Dr. Thackston agreed in the main with the writer, but stated that he had not accounted for the extreme sensitiveness often found on the line between the enamel and dentine, he believing that the connecting medium must be a vascular one, Dr. Wood's theory being that the sensitiveness arose from the terminal ramifications of the fibrillæ.

Dr. Thompson thought the report covered the entire accepted theory of the present day, and was sustained by microscopical researches.

Dr. Thackston, in proof of the views expressed by him, stated that if the source of supply and nutrition which existed between the dentine and pulp was severed, that the dentine would eventually darken and become a dead substance.

The president delivered his annual address, extending to all the members of the dental profession a hearty welcome, and announced as his theme, "The obligations of dentists to society, to the profession, and to each other." The progress of the profession was satisfactory, and our success most encouraging and gratifying. Dental surgery furnishes many examples of individual dignity and elevation of character, many conscientious and faithful observers of all the duties and obligations provided by the most rigid and exacting of ethical codes. Members we have who instinctively and from an innate sense of personal honor measure np in all their acts and transactions to that standard of moral and professional excellence that seeks no improper or selfish advantage, nor stops short of the fulfillment of every moral and professional obligation. We are required to be prompt in answering the calls and meeting the demands of our patients, to be tender and humane in our practice and ministrations, discreet and gentlemanly in our deportment, giving to our patrons the benefit of our highest skill. In no pursuit or profession of life is demanded a higher or more rigid illustration of all the qualities that distinguish a virtuous, refined and elevated gentleman, than in the relations and intercourse of the dental surgeon with his patrons and patients.

Our limited space forbids a more extended notice of the doctor's elegant and chastely written address, filled, as it was, with high and lofty sentiments and enforcing with high and commendable zeal the maxim, "Do unto others as you would have them do unto you."

### Afternoon Session.

Dr. Henkel read a very carefully prepared and interesting report on dental physiology. This subject had been in the past looked upon as of but little importance. So great has been the desire for active mechanical operating, that it is feared that many have by neglect of all physiological signs, so often met with in every-day practice, placed themselves altogether on a mechanical platform, and only able to look at their patients mechanically, closing their eyes to the physio-pathological fruit before them tempting them to pluck and digest mentally, thereby awaking out of the physiological sleep into which they have fallen. The report urged that a more rigid study of physiology be required of the dental student.

The report (of which we have only been able to give the concluding sentences) was discussed by Drs. Thackston, Wood, Moore, and others.

Dr. Thompson next read an admirable report on dental chemistry. That part of it which elicited the most discussion, was a reference to two important chemical agents, which, though of themselves old, were yet in their application new and highly useful in operative dentistry—namely: wood creosote and acetate of lead.

Dr. Wood advised a trial of the acetate of lead as recommended in the report.

Dr. Jeter believed that ood crweosote was a very different article from the creosote ordinarily used.

Dr. Thackston stated that creosote of commerce was only common carbolic acid.

Dr. Thompson advised the use of alcohol as an antidote for the escharotic effect of creosote.

Dr. Wood thought simple oil would answer the purpose.

Dr. Keach gave in detail an account of the application to the nerve of the sponge saturated in acetate of lead.

Dr. Wayt considered it well worthy the trial, and wondered that it had not occurred to him before, as in the early days of his practice he had used lead pivots, and always with success.

It is impossible, in the limits of a newspaper article, to do anything like justice to the very able, interesting, and instructive reports and discussions of this Association.

The Association adjourned to meet at 10 o'clock to-day.

### SECOND DAY.—Wednesday.

The meeting was called to order by the president at 10 o'clock, and the minutes of the preceding day read and approved.

The report on dental chemistry, the discussion of which was pending when the Association closed its previous session, was first taken up.

Dr. Burton recommended the application of carbolic acid simply to the nerve before capping with the oxychloride.

Dr. Mahoney had found no cause to regret his former practice of filling immediately the exposed nerve with the oxychloride, and expressed surprise at the statement made by some that such treatment had proved a failure.

Dr. Henkel had found that carbolic acid, applied in the manner described, was escharotic in its effects, and so the nerve was in a measure destroyed, and was afterwards likely to give trouble.

Dr. Hunt, of Washington, expressed gratification that the profession were giving more attention to chemistry than formerly. We should not only use chemical agents, but should know their properties, and how and and why they produce certain results. All art is based on science, and effects are the result of causes, and all subject to laws. We formerly destroyed nerves, now we seek to keep them alive, and these chemical agents are brought into requisition. We know not always why certain results follow, but we are progressing, and should not be satisfied until we can say we know; and in order to attain this end we should pursue diligently the study of this most important branch of our profession.

The report of dental pathology and surgery was next called for, but in the absence of the Committee the subject was passed, Dr. Henkel stating that the chairman, Dr. Johnson, had prepared a very elaborate report, but objected to sending it as he could not be present.

The subject of dental therapeutics was also passed, on account of the absence of the chairman of the committee. The president said, not in any spirit of censure, but as a suggestion, that it would be well for each member of a committee to prepare a report, and not rely on the chairman, and hoped this would be done in future.

Dr. Jeter, on behalf of the Executive Committee, tendered the use of the offices of city members of the Association for clinical exhibitions.

In reply to the query, What are the duties of the Executive Committee? the secretary read a portion of the constitution relating to that subject.

Dr. Steel suggested that they should also be made a committee on hospitalities, or that a special committee should at the proper time be appointed. It was desirable that there should be at all these meetings a full attendance. Especially should inducements be offered to those at a distance.

A communication was received from Dr. N. M. Burkholder, of Harrisonburg, Va.

On motion of Dr. Steel, the paper was referred to the president, with the request that if approved by him, it should be read under the head of Voluntary Essays.

Dr. Steel, chairman of the committee on mechanical dentistry, made a report on that subject. He reviewed in brief the qualities of the several bases used for mounting artificial teeth, was still of the opinion that there is not, never has been, nor ever will be, anything *superior* to gold for this object, but that the demand for something cheap was so great that the profession had been obliged to look around them for some material less expensive than gold or platina. Rubber had been and still was extensively used, but was objectionable on many accounts. Celluloid was now occupying the attention of the profession, and promised better results. He had been successful in its use, especially under the new method of preparing it by dry heat, and was glad to state that Dr. Hunt, the inventor of the dry heat process of preparing celluloid, was present, and no doubt prepared to give information on this subject.

Dr. Moore was pleased with the celluloid, and believed it would eventually supercede rubber.

Dr. Henkel, of Staunton, said that though he lived near where they kept crazy people, yet, as he was now among sane ones, he wanted to talk a little. He had used celluloid first with olive oil, then with tallow, but had not tried the dry heat referred to in the report; but from that and the subsequent discussion believed it to be the best cheap base now in use, though he had found difficulty in shaping it. Had also known it to assume a leathery consistency.

Dr. Hunt, the inventor of manipulating celluloid by the dry heat process, stated that celluloid could be *coaxed* to any desired shape, but could not be unduly forced. This forcing process doubtless caused the leathery consistency alluded to by Dr. Henkel. Celluloid is a new article; is made of cotton converted into gun cotton; then by the use of camphor into what is called pyroxyline. Hemp was in some cases substituted for cotton. Dr. Steel was correct in saying that *gold* was the best, and in recommending celluloid. It was best as compared with rubber, and it had some qualities superior to gold in that gold and silver felt hard in the mouth. In the natural teeth the periosteum counteracted the pain that would otherwise be occasioned by the occlusion of the teeth, so the springing, yielding properties of the celluloid served somewhat the purposes of nature in this respect. Celluloid is new; it is not yet four years since it was first introduced. He did not claim for it perfection, nor that it would supercede gold directly, but as rubber has to a large extent pushed gold out of the way, so he believed celluloid was destined to take the place of rubber, as the demand was for some cheap base. We know that rubber has been used under protest, particularly on account of its tendency to cause inflammation of the parts of the mouth with which it comes in contact. More can be done with celluloid, while its use is not injurious to the mouth. It approaches nearer in its nature and character the demands of the tissues. Celluloze, from which celluloid is made, is composed of carbon, hydrogen and oxygen. He thought the only objectionable feature about celluloid was its coloring matter, though that was slight as compared with rubber, for while rubber has 30 per cent. of vermillion, celluloid has only about one or one and a half per cent. Water, oil, steam, glycerine, and the dry heat had been successively used in manipulating the plate. He stated the advantages and disadvantages of each, and was satisfied that the new process of dry heat was the best. The camphor is a latent solvent, is volatile, and is evaporated by the heat leaving the celluloid seasoned. He exhibited a partial set warped by reason of not being seasoned, or by an unequal evaporation of the solvents. Described at length how these defects might be remedied. Was now directing his attention to the more thorough preparation of plaster of paris for making the moulds on which to shape the celluloid plates. (Dr. Mahoney inquired if all the camphor was evaporated would not the remaining mass disintegrate?) Dr. Hunt, resuming, said it was best not to drive out all of the camphor. After moulding the plate drive out as much of the camphor as is necessary. This can best be done by dry heat. This should not be done at too high a temperature, and as proof of it exhibited a spoilt piece caused by heating to too great an extent. A thermometer could not be used, but must be regulated by the judgment of the manipulator, or as had been expressed, by "rule of gumption"—common sense.

The hour of adjournment having arrived, on motion, Dr. Hunt was invited to give an exhibition of his process during the afternoon session.

### Afternoon Session.

The Association met promptly at 4 P. M.

Dr. L. M. Simms, of Chesterfield, and Dr. W. T. Downer, of King William, were elected to active membership.

Dr. Hunt now proceeded to exhibit his dry heat process of working the celluloid, and making such verbal explanations as were demanded of him. The members all seemed interested, and were doubtless instructed, as a unanimous vote of thanks was tendered him by the Society.

Dr. Steel exhibited a remarkable specimen of osseous union of the fangs of second superior molar and wisdom teeth which he had removed for a patient; also, some deciduous teeth which were filled seven or eight years ago, and remained perfect until they were shed, thus demonstrating the advantage of filling children's teeth, of which he is a great advocate, believing as he does that nature never designed that these teeth should be lost until the new ones were ready to take the place of them.

The Association then adjourned to meet at 10 o'clock Thursday, when the subject of mechanical dentistry will be resumed.

(TO BE CONTINUED.)

